# Modulating Backing Compliance for Low-Voltage, High-Strength Electroadhesive Gripping

**DOI:** 10.3390/polym18192358

**Published:** 2026-09-28

**Authors:** Jianping Wang, Shixiao Wang, Tianqi Pang, Min Gong, Xiang Lin, Fengxian Gao, Liang Zhang, Dongrui Wang

**Affiliations:** School of Chemistry and Biological Engineering, University of Science and Technology Beijing, Beijing 100083, China; b20200402@xs.ustb.edu.cn (J.W.); d202410502@xs.ustb.edu.cn (S.W.); d202510549@xs.ustb.edu.cn (T.P.); gongmin@ustb.edu.cn (M.G.); linxiang@ustb.edu.cn (X.L.); gaofx@ustb.edu.cn (F.G.); zhangliang@ustb.edu.cn (L.Z.)

**Keywords:** electroadhesion, conformal contact, electromechanical coupling, soft backing, low-voltage actuation

## Abstract

Coplanar electroadhesive (EA) devices present significant advantages for soft robotics and adaptive manipulation; however, achieving high adhesion forces at low operating voltages remains a critical challenge, primarily due to microscale interfacial air gaps that severely attenuate electrostatic attraction. While prior research has predominantly focused on dielectric material selection and electrode geometries, the crucial role of structural compliance, particularly that governed by the backing encapsulation layer, has been largely underestimated. Here, we introduce a flexible tri-layer EA pad incorporating a low-modulus polydimethylsiloxane (PDMS) backing layer. Upon application of an electric field, the induced Maxwell stress triggers localized elastic deformation within the soft backing layer, thereby enabling the ultrathin polyimide (PI) dielectric layer to seamlessly conform to the surface topographies of adherents. This “electro-induced conformal contact” mechanism dramatically reduces interfacial air gaps and shortens the effective electrostatic interaction distance. Under optimized conditions, the EA device achieves a robust 0.2 kPa normal adhesion at an ultralow driving voltage of merely 25 V and 112.7 kPa at 750 V. This work establishes a fundamental paradigm for safe, low-power robotic manipulation.

## 1. Introduction

Electroadhesion (EA) has emerged as a compelling, energy-efficient technology for controllable adhesion, finding diverse applications in soft robotics, adaptive gripping, bio-inspired climbing platforms, and human–machine haptic interfaces [1,2]. Operating through electrostatic forces generated by localized high-voltage electric fields, EA devices can adhere reversibly to a vast range of conductive and insulating substrates irrespective of their intrinsic magnetic or chemical properties via electrostatic induction and dielectric polarization [1,3]. A conventional planar electroadhesive device typically features a three-layer architecture: a thin dielectric contact layer designed to interface with the target substrate, a conductive electrode layer (commonly configured as coplanar interdigital electrodes to maximize electric field density), and a protective backing layer that encapsulates the electrodes to prevent electrical breakdown and provide structural support [3,4,5].

Historically, efforts to enhance electroadhesive performance have predominantly focused on optimizing the active interfacial components. Extensive research has investigated dielectric materials with high relative permittivity and breakdown strength [6,7,8], as well as variations in interdigital electrode geometries [4,9,10,11]—such as adjusting electrode width, spacing, and perimeter length. However, the role of the non-contact background encapsulation layer has largely been treated as a passive mechanical housing or merely an insulating barrier [5]. This approach overlooks the critical structural coupling between the backing layer and the rest of the device, which profoundly influences the pad’s overall compliance and mechanical behavior during contact.

A fundamental challenge limiting real-world electroadhesion capacity is the ubiquitous presence of interfacial air gaps [4,12,13,14,15]. Since electrostatic attraction decreases steeply with increasing effective separation, even micro-scale air gaps caused by surface roughness, waviness, or misaligned contact drastically degrade the net attractive force [12,13,14,15]. While dielectric compliance is known to aid surface adaptation [14,16], the device’s overall flexural rigidity and deformation response are strongly dictated by the backing layer due to its relative thickness. Consequently, the thickness and elastic modulus of the backing layer govern the device’s apparent flexural modulus. A poorly tailored backing layer can impose excessive stiffness, inhibiting full interfacial intimacy and leading to larger air gaps and severe loss of electroadhesive performance. Conversely, optimizing these mechanical parameters enables the device to conform seamlessly under the action of electrostatic forces.

In this work, we systematically investigate how the thickness and elastic modulus of the backing layer affect the electroadhesive capacity of tri-layer EA devices. We demonstrate that the normal adhesion force exhibits a nonlinear, inverted U-shaped trend with independent variation in PDMS thickness and modulus. By establishing this optimized parameter window, we further unveil the underlying dynamics of the electro-induced conformal contact mechanism: the initial electrostatic attraction acts as a precursor force that compresses the soft backing and minimizes interfacial micro-cavities. Validated by in-situ contact impedance (ΔC) measurements and interface optical observations, the device proposed in this study is capable of achieving effective electroadhesion at relatively low voltages (0.2 kPa at 25 V) and can reach significantly high adhesion strengths at medium-to-high voltages (112.7 kPa at 750 V) (Table S1, [17,18,19,20,21,22]). This work not only establishes a quantitative framework for incorporating encapsulation mechanics into electrostatic dynamics but also uncovers the critical role of modulus mismatch in multilayered heterogeneous structures, thereby charting a clear course for the future design of highly robust, low-voltage electroadhesion interfaces.

## 2. Materials and Methods

***Fabrication of the EA pads.*** To construct the devices, a 4 × 6 cm^2^ glass slide was employed as a rigid carrier to support a 5 μm-thick PI membrane (Kaneka, Japan) (Figure S1). Au interdigitated electrodes were subsequently deposited onto the polymer surface using an ion sputter coater (GVC 2000, Hezao, Shanghai, China). The specific electrode geometry was defined by a bespoke stainless-steel shadow mask, creating a 1.06 cm^2^ active region comprising 730-μm-wide lines separated by 168-μm gaps (Figure S2).

Next, a liquid precursor of PDMS (Sylgard 184, Dow-Corning, Midland, MI, USA) was prepared. For all investigated base-to-curing-agent ratios ranging from 5:1 to 50:1, the preparation and curing procedures were kept consistent, with only the ratio itself being varied. Specifically, all PDMS mixtures were vacuum-degassed for 10 min to remove air bubbles before blade coating. Subsequently, the mixture was spread over the patterned substrate via blade coating to form the soft elastomeric backing. This layer was initially crosslinked in an oven at 70 °C for 30 min. Afterward, the complete assembly was carefully peeled from the temporary glass support and underwent a full thermal post-cure at 70 °C for 24 h to guarantee long-term structural integrity.

By systematically modifying the blade clearance during the coating phase—specifically to 250, 500, 750, 1000 and 1250 μm—we successfully generated a series of EA actuators with varying backing thicknesses.

***Measurements of interfacial adhesion.*** To evaluate the normal adhesive performance, a customized arrangement was specifically designed to handle the mechanical compliance of the soft thin-film structures, allowing for precise physical alignment throughout the dynamic testing process. The EA pad was anchored to a rigid glass platform, orienting the PDMS backing downwards so that the active PI dielectric layer remained exposed. A 2.0 × 2.0 × 2.0 cm^3^ glass block, functioning as the target adherend, was attached to a universal mechanical tester (ESM 303, Mark-10, Bohemia, NY, USA) and programmed to descend onto the EA surface. The glass block was connected to the force sensor via a soft string. All measurements took place under stable ambient conditions (room temperature, 37–45% relative humidity) using a high-voltage direct current source (Model 2290-10, Keithley, Solon, OH, USA) for actuation.

Baseline calibrations were conducted by lowering the glass block onto the unpowered EA pad for 5 min. After factoring in the adherend’s intrinsic mass, the baseline mechanical adhesion at 0 V was confirmed to be negligible. The adherend’s weight was approximately 3.15 g, corresponding to a preload of 0.309 kPa. During active testing, the glass block was brought back into contact, and a predefined DC voltage was supplied for a 5-min dwell period. The glass block was then withdrawn vertically at a steady rate of 13 mm/min. The effective normal electrostatic force was derived by subtracting the block’s gravitational weight from the absolute maximum force registered upon detachment. To prevent history effects between trials, the contact interfaces were wiped clean with ethanol, and any accumulated surface charges on the EA pad were neutralized by either grounding the system or applying a low-voltage AC signal for 10 min. Surface roughness data for both the EA pad and various adherends were also provided in Table S2. For electroadhesion tests under different PDMS thickness and modulus conditions, at least three independent EA devices were used for each parameter set. Adhesion force data for each voltage condition were obtained through at least three repeated measurements, ensuring no fewer than three valid independent data points were collected.

***Electrical and impedance characterization.*** Operating fundamentally as a coplanar interdigitated capacitor, the energy dissipation of the device was quantified by capturing both its transient and steady-state leakage currents. This characterization was performed by wiring a precision electrometer (Model 6514, Keithley, USA) in series with a specialized high-voltage DC power source.

To continuously track the dynamic evolution of the physical contact interface, the testing apparatus was isolated within an electrostatic shielding enclosure and probed using an impedance analyzer (TH2829C, Tonghui, Changzhou, Jiangsu, China). While the primary high-voltage DC excitation was applied directly across the positive and negative terminals of the interdigitated electrodes to induce electroadhesion, the impedance analyzer was simultaneously bridged between the negative electrode terminal and the conductive target object (an iron block) to monitor the real-time interfacial sealing behavior.

***TIR imaging methodology.*** As depicted in Figure S3, the EA pad was secured onto a glass substrate via wet adhesion. A transparent glass block with a side length of 1 cm was naturally placed over the electrode region of the EA pad. A camera, positioned obliquely downward from the side of the glass block, continuously recorded the contact interface between the glass block and the EA pad. This recording captured the process where a DC power supply delivered 500 V to the EA pad for 1 min before being disconnected. The frame captured at the 1-min mark (just before power disconnection) was extracted for TIR image analysis.

## 3. Results

### 3.1. Device Architecture and Electro-Induced Conformal Contact Mechanism

In traditional rigid EA systems, the microscopic roughness of both the EA pad and the target adherend hinders ideal interfacial intimacy, resulting in residual air gaps that severely attenuate electrostatic attraction. To achieve high electroadhesion strength at lower driving voltages, we designed and fabricated a flexible coplanar EA device featuring a three-layer architecture: an ultrathin polyimide (PI) dielectric film (relative permittivity ε_r_ ≈ 3.4, thickness t = 5 μm, Young’s modulus E = 5.23 ± 0.35 GPa, Figure S4), sputtered gold (Au) interdigitated electrodes (thickness t ≈ 25 nm, Figure S5), and a polydimethylsiloxane (PDMS) encapsulation/backing layer from top to bottom (Figure 1a). This PI-Au-PDMS design simultaneously lowers the required operating voltage and enhances macroscopic adhesion performance.

The core mechanism underlying this enhancement is the “electro-induced conformal contact” process, which is enabled by the layered structure (Figure 1b). In the unpowered state (voltage off), microscopic interfacial air gaps inevitably persist between the device and rigid adherend. Upon applying a driving voltage, Maxwell stress is generated across the dielectric interface, which induces localized elastic deformation within the soft PDMS backing layer. This soft backing acts as a compliance buffer, enabling the ultrathin PI layer to seamlessly bend and conform to the micro-topography of the target surface. As a result, the initial rigid point contacts transition into an intimate, conformal interface. This structure reconfiguration greatly reduces interfacial air gaps and shortens the effective electrostatic interaction distance, thereby boosting interfacial electroadhesion. By systematically tuning the thickness and stiffness of the PDMS layer, the optimized device (using the PDMS layer, which was blade-coated at a gap of 500 μm with a 20:1 base-to-curing-agent ratio) exhibited excellent low-voltage, high-adhesion characteristics. As shown in Figure 1c, within a DC voltage of 25 V to 750 V, the electroadhesion strength in the normal direction scaled dramatically from 0.2 kPa to 112.7 kPa, demonstrating the efficacy of modulating structural compliance to maximize interfacial electrostatic coupling.

### 3.2. Parametric Modulation of the Compliant Backing Layer

In multilayer flexible EA devices, the geometric dimensions and mechanical properties of the underlying PDMS backing layer critically govern the macroscale electro-actuation and adhesion performance. As illustrated in Figure 2a, a custom measurement setup was configured to quantify the normal electroadhesion strength. An inverted testing configuration with the EA device facing downward and a glass test block approaching from the top was implemented to ensure precise spatial alignment and prevent gravitational sagging of the soft, large-area thin films, thereby minimizing systematic measurement errors.

Under a constant driving voltage of 500 V with all other parameters fixed, we systematically investigated the effects of the PDMS layer’s thickness and Young’s modulus on the resulting adhesion strength. As depicted in Figure 2b and Table S3, the normal electroadhesion strength exhibits a distinct non-monotonic, inverted U-shaped dependence on the PDMS layer’s thickness. This behavior was mechanistically corroborated by total internal reflection (TIR) optical characterization at the contact interface (Figure 2c). Following a 1 min voltage application at 500 V, the gradual elimination of interfacial air gaps leads to a pronounced variation in optical contact quality: the electrode pattern evolves from fragmented to crisp and fully developed, before blurring at excessive thickness, reaching an optimal conformal contact state at a thickness of 500 μm. This confirms a trade-off mechanism wherein an overly thin PDMS layer lacks sufficient compliance to absorb micro-topographical asperities, whereas an excessively thick PDMS layer imparts unwanted flexural rigidity that suppresses local interfacial deformation.

To quantitatively assess the degree of interfacial conformity, the optical micrographs were processed using MATLAB (MATLAB, R2026a, MathWorks, Natick, MA, USA). First, the raw RGB images were converted into 8-bit grayscale images. Subsequently, the images were binarized by applying Otsu’s adaptive thresholding method to extract the effective contact area ratio, which we termed “contact completeness”. Furthermore, the optical contact intensity was quantified by calculating the inverted average grayscale value within these specific thresholded masks, referred to as the “optical intensity score”. These quantitative metrics (as shown in Figure S6) further supported the visual observations from the TIR images and provided numerical evidence for the optimal conformal contact state. For instance, Figure S6a illustrates the contact completeness and optical intensity score as a function of blade height, demonstrating optimal geometric conformity at a height of 500 μm.

Similarly, the cross-linker ratio, which dictates the Young’s modulus of the resultant PDMS, plays a crucial regulatory role. Tensile testing confirms that increasing the base-to-curing-agent ratio drastically softens the polymer network, leading to a marked decrease in Young’s modulus (Figure 2d). Consequently, the normal adhesion strength displays a similar inverted U-shaped trend as a function of the mixing ratio under a driving voltage of 500 V (Figure 2e). The corresponding TIR micrographs (Figure 2f) further reveal that, at a fixed coating gap of 500 μm, base-to-curing-agent ratios of 20:1 and above produce the most continuous contact footprint with maximum optical clarity. Through quantitative analysis (as shown in Figure S6b), we found that the contact completeness rapidly saturates at a 20:1 ratio, while the optical intensity score exhibits local maxima at 20:1 and 50:1, indicating exceptionally tight localized contact for these specific formulations. These results indicate that while lowering the elastic modulus enhances local interfacial adaptation, an excessively soft backing layer (e.g., 50:1) induces adverse bulk viscoelastic dissipation and insufficient elastic recovery force, ultimately compromising the net macroscopic adhesion capacity.

### 3.3. In-Situ Validation of Interfacial Gap Closure

To dynamically probe the electro-induced conformal evolution of the contact interface during activation, we developed an in-situ contact impedance measurement platform (Figure 3a). The corresponding equivalent circuit (Figure 3b) illustrates the decoupled electromechanical configuration of our system: the high-voltage DC source exclusively actuates the upper domain (the interdigital electrodes), while the AC impedance analyzer monitors the lower interfacial domain (comprising PI film and the interfacial air gap). Because the PI dielectric thickness remains invariant, the measured series capacitance (C_s_) serves as a sensitive, direct probe for real-time air gap closure (ΔC).

It is noteworthy that a conductive iron block was chosen as the adherend for impedance measurements to ensure a complete electrical circuit with the impedance analyzer. Although the adherend type differs from the glass block used in normal adhesion force tests, we confirmed the consistency of the electro-induced conformal contact mechanism in both scenarios through supplementary experiments. Figure S7 demonstrates that the normal adhesion force exhibits an inverted U-shaped trend with PDMS thickness when using a conductive iron block, which is highly consistent with the results obtained using a dielectric glass block. This indicates the universality of the regulatory mechanism of PDMS backing layer compliance on interfacial air gap closure and increased actual contact area.

The dynamic step-response profiles (Figure 3c,d) reveal an abrupt, step-like increase in C_s_ immediately upon voltage application, confirming the rapid squeeze-out of interfacial air pockets under the induced Maxwell stress. By extracting steady-state capacitance values across different operational states (0 V initial, 500 V actuating, and 0 V relaxed post-actuation), we plotted the steady-state capacitance variations against the PDMS thickness (Figure 3e) and Young’s modulus (Figure 3f). Remarkably, this interfacial capacitance mapping faithfully mirrors the inverted U-shaped profiles observed in the macroscale mechanical test (Figure 2b,e). This striking quantitative alignment provides direct evidence for the electro-induced conformal contact mechanism: an optimized soft backing yields under electrostatic forces to flatten the PI layer and squeeze out interfacial air gaps, maximizing the active contact capacitance and ultimately translating into peak macroscale normal electroadhesion. This mutual corroboration unequivocally establishes that modulating the thickness and modulus of the PDMS backing layer directly dictates interfacial air gap, serving as the governing origin of adhesion strength enhancement in flexible EA devices.

Further theoretical support for the electro-induced conformal contact mechanism is provided by finite element method (FEM) simulations. We developed a mechanical force model where the electrostatic attraction generated by electrification was simulated by applying an equivalent downward displacement or pressure to the interface. The simulation results, presented in Figure S8, clearly illustrate how the soft PDMS backing layer undergoes localized elastic deformation under this simulated external pressure, enabling the overlying PI dielectric layer to conform better to rough surfaces and significantly reduce interfacial air gaps. These FEM simulations offer a valuable theoretical perspective, complementing our experimental observations and reinforcing the understanding of the underlying physical processes.

### 3.4. Conceptual Mechanical Model for the Inverted U-Shaped Dependence

To gain a deeper understanding of the inverted U-shaped dependence of electroadhesion strength on PDMS backing layer thickness ($t_{PDMS}$) and Young’s modulus ($E_{PDMS}$), we propose a conceptual mechanical model framework. This framework aims to analyze and elucidate the underlying mechanisms. Upon voltage application, Maxwell stress (*P_M_*) is generated between the electroadhesive device and the adherend, driving the deformation (*δ*) of the PDMS backing layer. The compliance (*κ*) of the PDMS backing layer is considered to be inversely proportional to its Young’s modulus and directly proportional to some power of its thickness ($\kappa \propto 1/\left(E_{PDMS}t_{PDMS}^{n}\right)$, where n≥1). The deformation *δ* is approximately proportional to $P_{M}\cdot \kappa$.

Initial microscopic air gaps are reduced by the deformation of the PDMS backing layer, thereby increasing the actual contact area ($A_{contact}$). $A_{contact}$ can be expressed as $A_{contact}=A_{initial}+(A_{max}-A_{initial})\cdot f(\delta )$, where $A_{initial}$ is the initial contact area, $A_{max}$ is the theoretical maximum contact area, and f(δ) is a monotonically increasing function with saturation characteristics. The final electroadhesion force ($F_{adhesion}$) is proportional to $A_{contact}$.

This conceptual model explains the observed inverted U-shaped trend: (i) Excessively high PDMS modulus or low thickness (*κ* too small): The backing layer is too rigid, resulting in minimal deformation δ, and thus $A_{contact}$ remains close to $A_{initial}$, leading to low adhesion force. (ii) Optimized PDMS modulus or thickness (*κ* moderate): As *κ* increases, deformation δ increases, and $A_{contact}$ significantly expands, causing the adhesion force to rise rapidly and reach a peak. This corresponds to our proposed “electro-induced conformal contact” mechanism. (iii) Excessively low PDMS modulus or high thickness (*κ* too large): Although theoretically increased compliance might lead to greater deformation, physical limitations such as viscoelastic dissipation, insufficient elastic recovery force, and reduced overall structural stability cause the adhesion force to decrease.

This model framework highly correlates with the inverted U-shaped trends observed in adhesion force (Figure 2b,e) and capacitance (Figure 3e,f). The increase in capacitance directly reflects the reduction in interfacial air gaps and the increase in actual contact area, precisely as predicted by this model. When the compliance of the PDMS backing layer is within the optimized window, the electric field-induced deformation maximizes the actual contact area, thereby achieving the highest electroadhesion force.

### 3.5. Versatile Applications and Energy Efficiency

Beyond characterizing interfacial contact mechanics and capacitive responses, capturing the transient electrical current behavior during activation and detachment cycles is imperative for quantifying energy consumption and operational safety. Figure 4a,b display the transient current profiles of the optimized EA device under two representative operational sequences: de-energization prior to mechanical detachment (Figure 4a), and mechanical detachment under active bias followed by voltage shut-off (Figure 4b). Upon instantaneous voltage application, a nanoampere (nA)-level displacement current spike emerges in the circuit, originating from the rapid charging of the equivalent dielectric capacitance alongside the abrupt capacitance increment induced by interfacial air gap closure. During the voltage-holding phase, the current rapidly decays to an ultralow leakage steady state, with power consumption not exceeding 0.044 mW. The instantaneous peak power of the electroadhesive device does not exceed 0.142 mW (Table S4) at an operating voltage of 500 V.

Benefiting from the simple tri-layer architecture and superior electro-induced conformal contact capabilities, the engineered EA device demonstrates exceptional versatility and reliability in practical applications. As showcased in Figure 4c, the device demonstrates both high-capacity normal lifting and gentle, tangential manipulation, enabling the damage-free engagement of fragile objects with complex geometries, such as raw eggs—a feat challenging for conventional rigid suction cups. Furthermore, integrating this conformable EA pad onto a robotic end-effector provides a versatile adhesion interface, significantly improving the grasping stability and success rate when manipulating low-friction or high-curvature substrates (e.g., smooth glass bottles). These findings collectively highlight the immense potential of tailoring backing compliance for next-generation electroadhesive technologies, spanning soft robotic end-effectors, non-destructive material handling, and adaptive wearable systems.

## 4. Discussion

The widespread application of coplanar EA devices has long been hindered by the necessity of high driving voltages. In this study, we addressed this critical challenge by shifting the structural optimization paradigm from traditional electrode layout design to the mechanical modulation of the encapsulation layer. We developed a flexible tri-layer EA device, wherein a low-modulus PDMS backing layer functions as a dynamic compliance buffer to actively regulate interfacial electromechanical coupling.

Our systematic investigation reveals that the macroscale normal electroadhesive performance is largely governed by the thickness and elastic modulus of the soft backing layer, exhibiting a distinct inverted U-shaped parametric design window. This structural tuning explicitly elucidates the mechanics of the electro-induced conformal contact process: initial electrostatic attraction acts as a precursor force to deform the compliant backing, which in turn enables the ultrathin PI film to seamlessly conform to target surface topographies. This localized deformation effectively eliminates interfacial micro-cavities and shortens the electrostatic interaction distance, thereby maximizing macroscopic adhesion capacity. By operating within the optimized structural window, the device achieves effective electroadhesion at relatively low voltages (0.2 kPa at 25 V) and significantly high adhesion strengths at medium to high voltages (112.7 kPa at 750 V).

Despite the promising results and the inclusion of additional testing data on various substrates (Table S4), we acknowledge certain limitations of this study. Specifically, electroadhesion performance is highly dependent on the material properties and surface topography of the adherend. While our optimized design demonstrates improved performance across different surfaces, a comprehensive understanding and predictive model for diverse substrate interactions remain areas for further investigation.

Ultimately, this work provides a fundamental experimental and analytical framework for designing low-voltage, high-efficiency soft electroadhesive interfaces, paving the way for versatile robotic manipulation and safe human–machine interactions. However, a structural limitation inherent in this multilayer design must be objectively acknowledged. Due to the extreme elastic modulus mismatch between the relatively rigid PI dielectric layer (~GPa) and the soft PDMS backing layer—spanning several orders of magnitude—severe stress concentration occurs at the heterogeneous interface during normal pull-off separation under high adhesion forces. This mismatch makes the device susceptible to irreversible delamination and peeling failure over time. Consequently, addressing this mechanical vulnerability provides a clear avenue for future research, where constructing toughened, chemically bonded, or more continuous gradient-modulus interfaces will be crucial for the durability and practical deployment of next-generation soft electroadhesive systems.

## Figures and Tables

**Figure 1:**
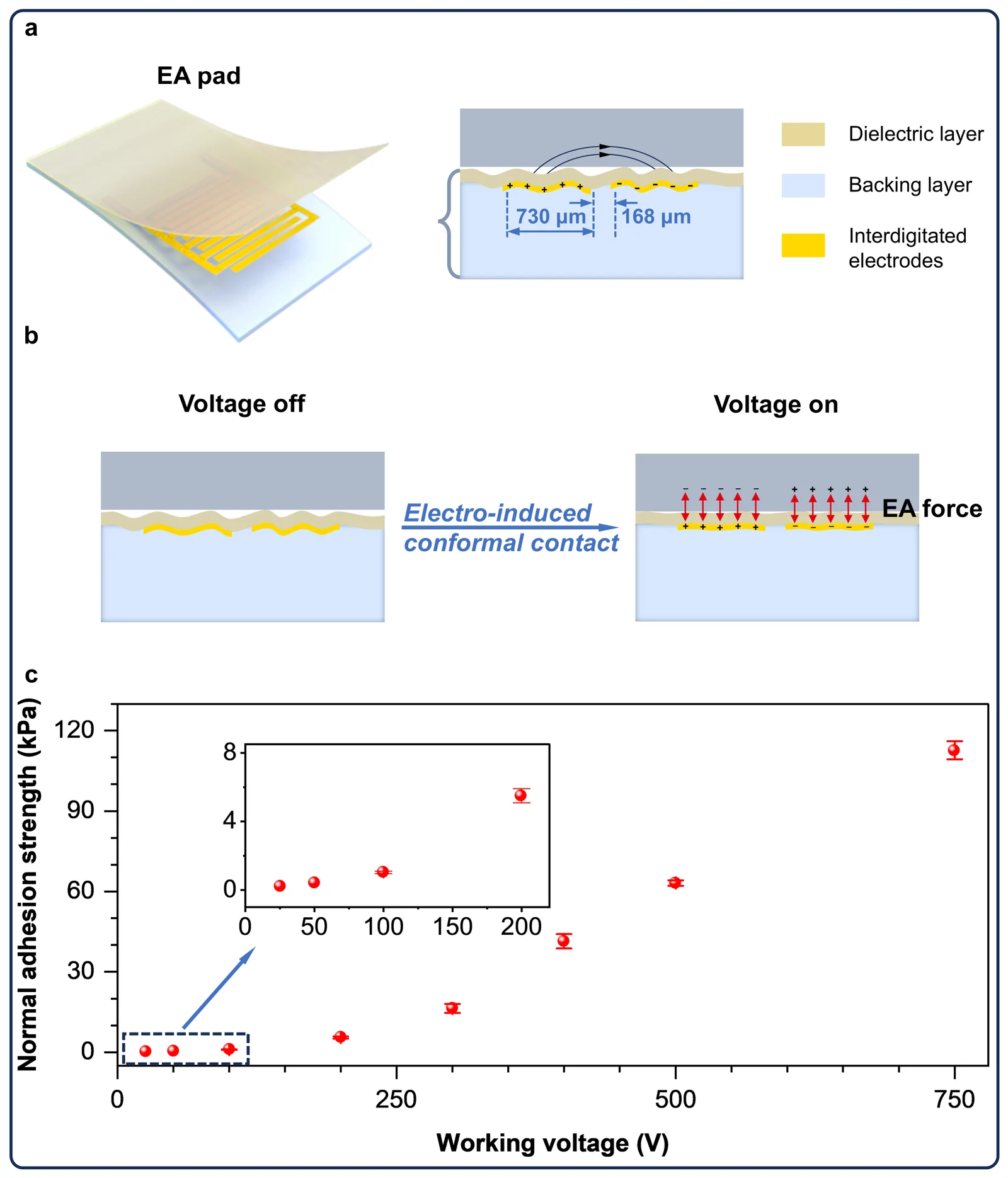
Device architecture and electro-induced conformal contact mechanism for the flexible EA device. (**a**) Schematic illustration and cross-sectional parameters of the coplanar EA pad, comprising an ultrathin PI dielectric layer, Au interdigitated electrodes, and a PDMS backing layer. (**b**) Schematic of the electro-induced conformal contact transition from an initial state with interfacial air gaps to a conformal state, which reduces the gap and significantly enhances the EA interaction. (**c**) Normal adhesion strength as a function of driving voltage (25 V to 750 V) under the optimized PDMS encapsulation parameters (blade-coated at a gap of 500 μm, base-to-curing-agent ratio of 20:1).

**Figure 2:**
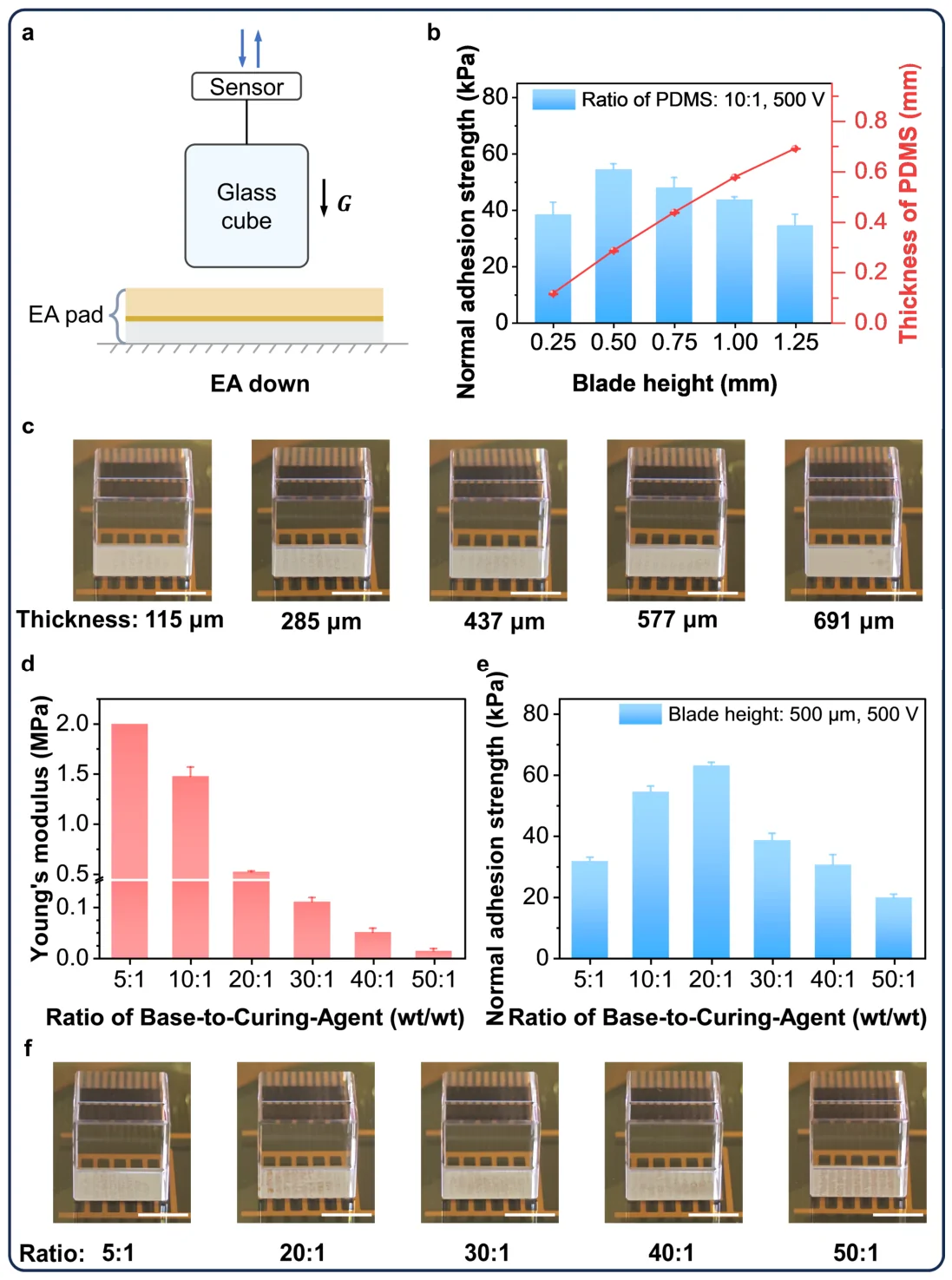
Regulatory effects of PDMS backing layer’s thickness and modulus on adhesion performance. (**a**) Schematic of the measurement setup for normal adhesion strength. (**b**) Normal adhesion strength and corresponding PDMS thickness as a function of the blade-coating height under a 500 V driving voltage (PDMS base: curing agent, 10:1). (**c**) TIR graphs at the contact interface under different blade-coating heights after applying 500 V for 1 min (PDMS base: curing agent, 10:1). (**d**) Young’s modulus of PDMS across different base-to-curing agent ratios. (**e**) Normal adhesion strength as a function of PDMS base-to-curing agent ratio at 500 V (blade-coating height of 500 μm). (**f**) TIR graphs at the contact interface under different PDMS base-to-curing agent ratios after applying 500 V for 1 min (blade-coating height of 500 μm). Scale bar in the photos: 1 cm.

**Figure 3:**
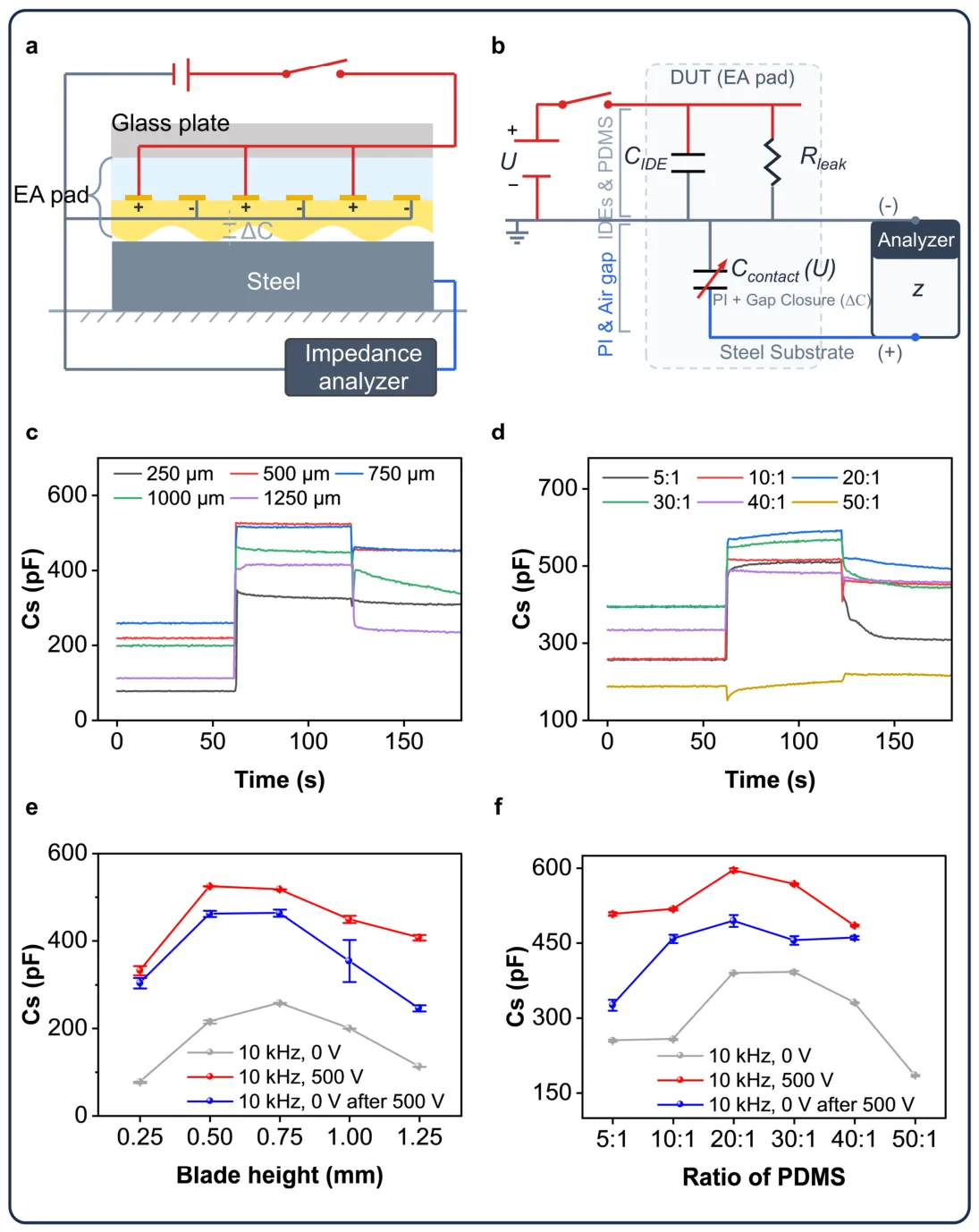
In-situ capacitance monitoring for the verification of interfacial air gap evolution. (**a**) Schematic of the in-situ contact impedance measurement platform. (**b**) Equivalent circuit model decoupling the DC high-voltage actuation domain and the AC impedance monitoring domain. (**c**,**d**) Dynamic step-response curves of the interfacial capacitance (C_s_) upon voltage application for varying PDMS (**c**) thicknesses and (**d**) mixing ratios (tested at 10 kHz). (**e**,**f**) Extracted steady-state capacitance versus PDMS (**e**) thickness and (**f**) Young’s modulus under different voltage states, explicitly mirroring the inverted-U trends observed in macroscale electroadhesion test.

**Figure 4:**
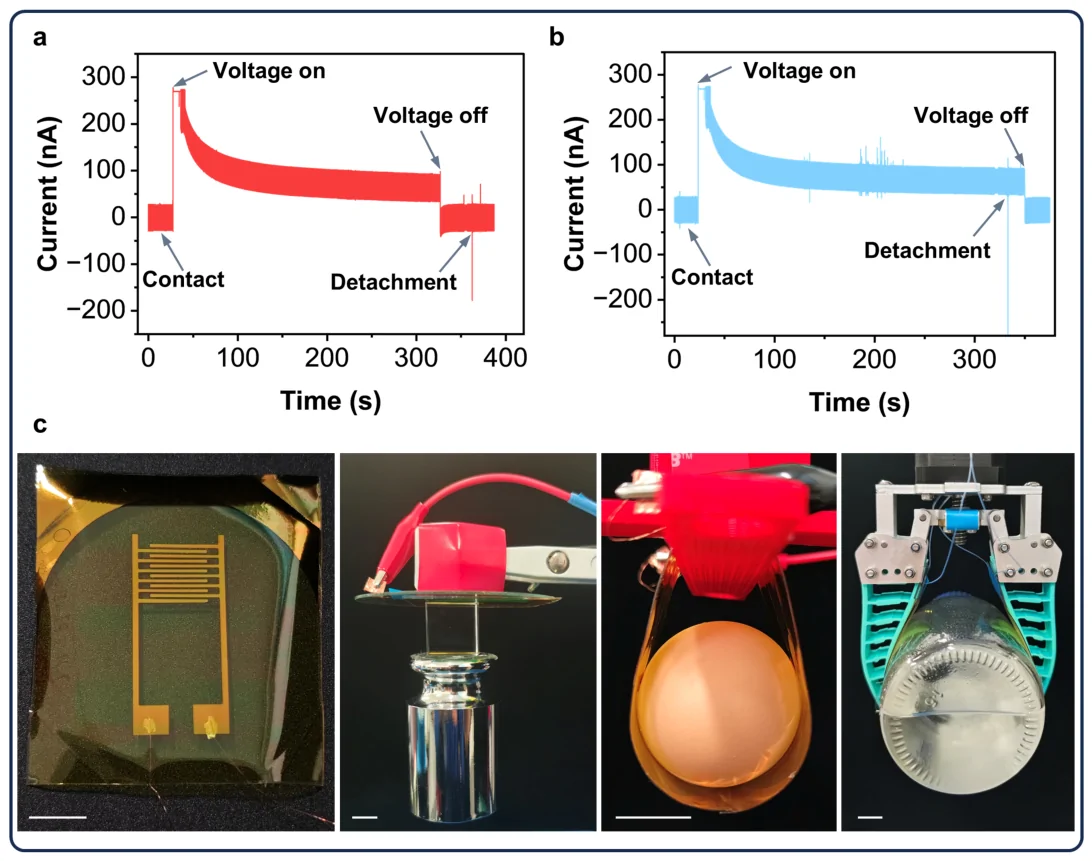
Transient current response and practical grasping applications of the EA devices. (**a**) Current variation in the EA device during a working cycle at 500 V with a “voltage off followed by detachment” sequence. (**b**) Current variation during a working cycle at 500 V with a “detachment followed by voltage off” sequence. (**c**) Photographs demonstrating practical application scenarios: using the tri-layer EA device for high-load normal lifting of a standard weight, non-destructive tangential grasping of a fragile egg, and integration with a mechanical gripper for the assisted grasping of a smooth glass bottle at 500 V. Scale bar in the photos: 1 cm.

## Data Availability

All data are available in the main text or the Supplementary Materials.

## Supplementary Materials

The following supporting information can be downloaded at: https://www.mdpi.com/article/10.3390/polym18192358/s1, Figure S1: Fabrication process of the EA pads. (a,b) A stainless-steel shadow mask is magnetically secured onto a 5-μm-thick polyimide (PI) film (Kaneka, Japan). (c) Au electrodes are deposited onto the PI dielectric via magnetron ion sputtering (GVC 2000, Hezao Lab, China). (d,e) Following mask removal, a PDMS backing layer (Sylgard 184, Dow Corning, USA) is blade-coated over the patterned surface and then crosslinked in an oven at 70 °C. (f) The finalized EA pad after delamination from the temporary glass substrate; Figure S2: Optical image of the Au electrode patterns; Figure S3: Schematic illustration of the Total Internal Reflection (TIR) imaging setup. This apparatus is utilized for real-time monitoring of the interfacial contact state between the electroadhesive device and the target substrate. By capturing light reflected from the interface with a high-resolution camera (1080 P, 30 fps), the formation and evolution of the contact area can be visualized and quantitatively analyzed; Figure S4: Tensile stress-strain curve of the 5-μm-thick PI film; Figure S5: AFM cross-sectional profile demonstrating a distinct physical step height of ~25 nm at the electrode edge; Figure S6: Quantitative TIR image analysis of interfacial contact. (a) Extracted contact completeness (black curve) and optical intensity score (red curve) as a function of blade height, demonstrating optimal geometric conformity at a height of 0.50 mm. (b) Dependence of the contact metrics on the PDMS mixing ratio. The completeness rapidly saturates at a 20:1 ratio, while the optical intensity score exhibits local maxima at 20:1 and 50:1, indicating exceptionally tight localized contact for these specific formulations; Figure S7: Normal adhesion strength under a 500 V driving voltage (PDMS base: curing agent, 10:1) with the adherend of steel; Figure S8: Finite Element Method (FEM) Simulation Analysis of the Electro-induced Conformal Contact Mechanism. **Top:** In the initial state with no applied voltage (Voltage off, no electrostatic force), significant air gaps are evident at the interface due to microscopic roughness. **Bottom:** Upon application of an equivalent electrostatic force (Voltage on), the soft PDMS backing layer undergoes substantial localized elastic deformation under the simulated pressure (indicated by the color-mapped stress field). This underlying elastic deformation effectively drives the conformal adaptation of the top PI dielectric layer to the rough surface, thereby largely eliminating interfacial micro-cavities. **Right:** High-magnification schematic diagrams visually compare the evolution of local microscopic topography and the physical closure of the interface before and after the application of the electrostatic field; Table S1: Performance comparison of the proposed EA device with low-voltage electroadhesive devices reported in literature; Table S2: Surface roughness of both the EA pad and various adherends; Table S3: Actual thicknesses of PDMS backing layers as a function of blade height and PDMS mixing ratio; Table S4: Electroadhesion force and instantaneous power of EA devices with varying parameters on different adherends at 500 V working voltage. (“10:1–0.25 mm” denotes the preparation parameters of the PDMS backing layer, “10:1” refers to a mixing ratio of 10 parts PDMS base to 1 part curing agent, and “0.25 mm” indicates that the blade height was set to 0.25 mm during the blade-coating process).

## Abbreviations

The following abbreviations are used in this manuscript:

EA	Electroadhesion
PDMS	Polydimethylsiloxane
PI	Polyimide
TIR	Total internal reflection
nA	Nanoampere
