# Supplementary material for: Modulating Backing Compliance for Low-Voltage, High-Strength Electroadhesive Gripping
Source: Polymers (Basel). 2026 Sep 28;18(19):2358. doi: 10.3390/polym18192358 (PMC13666949; doi:10.3390/polym18192358)
Supplement: Supplementary file 1 [file polymers-18-02358-s001.zip › polymers-4566278-supplementary.pdf]

SUPPLEMENTARY INFORMATION

Modulating Backing Compliance for Low-Voltage, High-Strength Electroadhesive Gripping

*Jianping Wang, Shixiao Wang, Tianqi Pang, Min Gong, Xiang Lin, Fengxian Gao, Liang Zhang, Dongrui Wang**

School of Chemistry and Biological Engineering, University of Science and Technology Beijing, Beijing, 100083, China

E-mail: wangdr@ustb.edu.cn

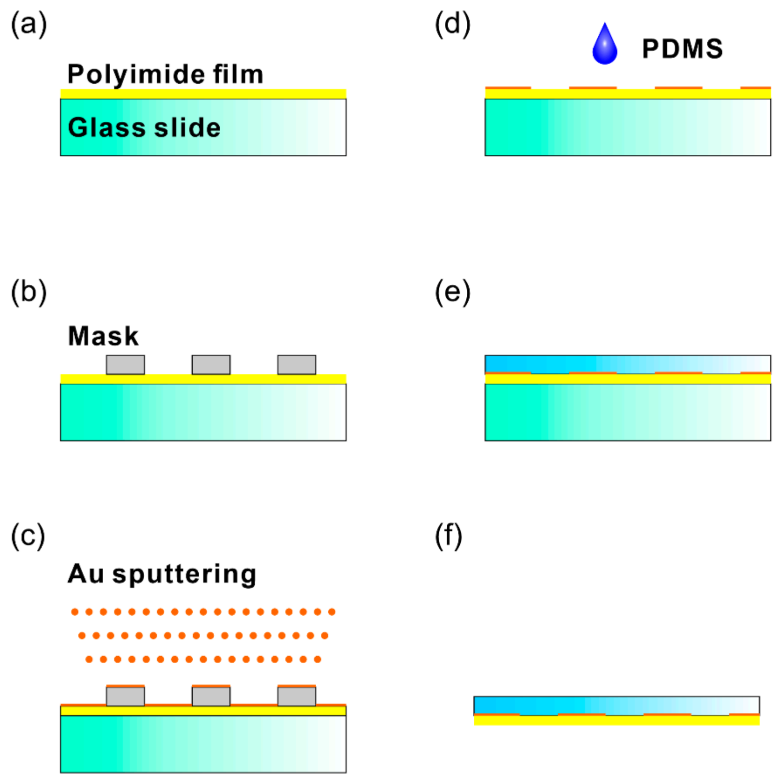

Figure S1. Fabrication process of the EA pads. (a-b) A stainless-steel shadow mask is magnetically secured onto a 5-μm-thick polyimide (PI) film (Kaneka, Japan). (c) Au electrodes are deposited onto the PI dielectric via magnetron ion sputtering (GVC 2000, Hezao Lab, China). (d-e) Following mask removal, a PDMS backing layer (Sylgard 184, Dow Corning, USA) is blade-coated over the patterned surface and then crosslinked in an oven at 70°C. (f) The finalized EA pad after delamination from the temporary glass substrate.

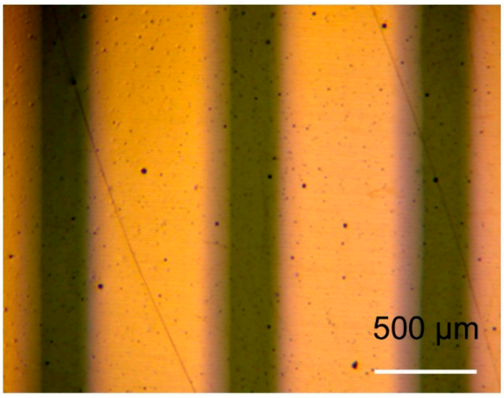

Figure S2. Optical image of the Au electrode patterns.

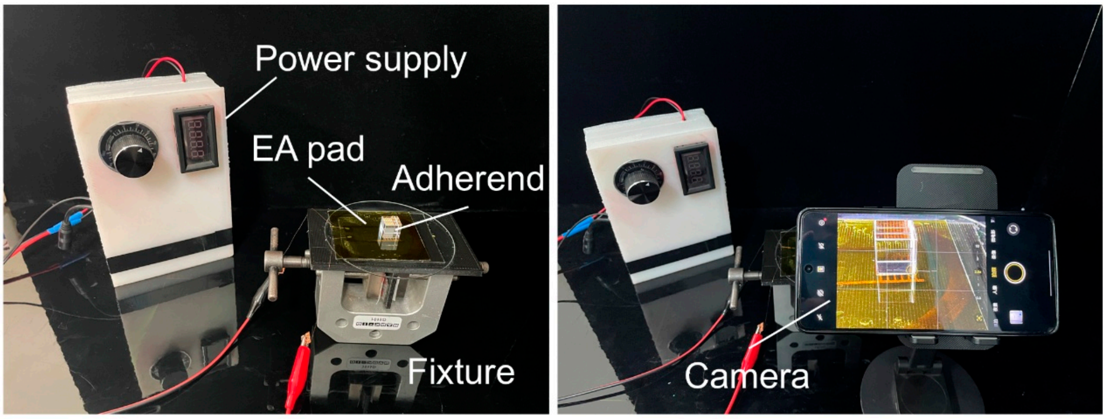

Figure S3. Schematic illustration of the Total Internal Reflection (TIR) imaging setup. This apparatus is utilized for real-time monitoring of the interfacial contact state between the electroadhesive device and the target substrate. By capturing light reflected from the interface with a high-resolution camera (1080 P, 30 fps), the formation and evolution of the contact area can be visualized and quantitatively analyzed.

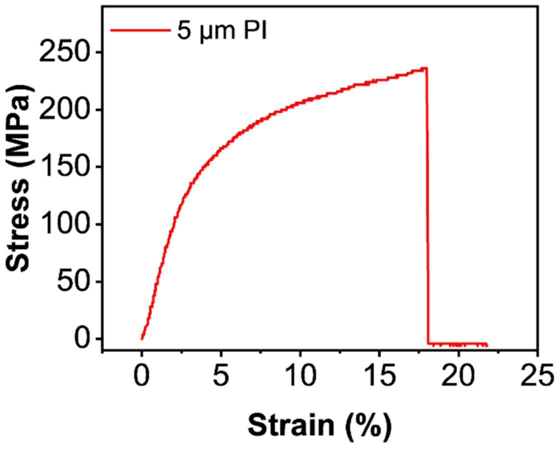

Figure S4. Tensile stress-strain curve of the 5-μm-thick PI film.

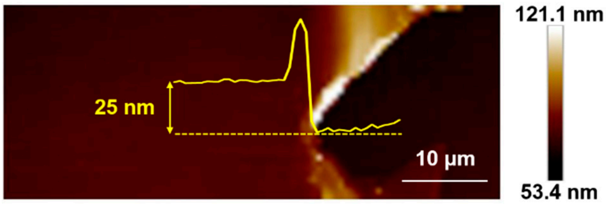

Figure S5. AFM cross-sectional profile demonstrating a distinct physical step height of ~25 nm at the electrode edge.

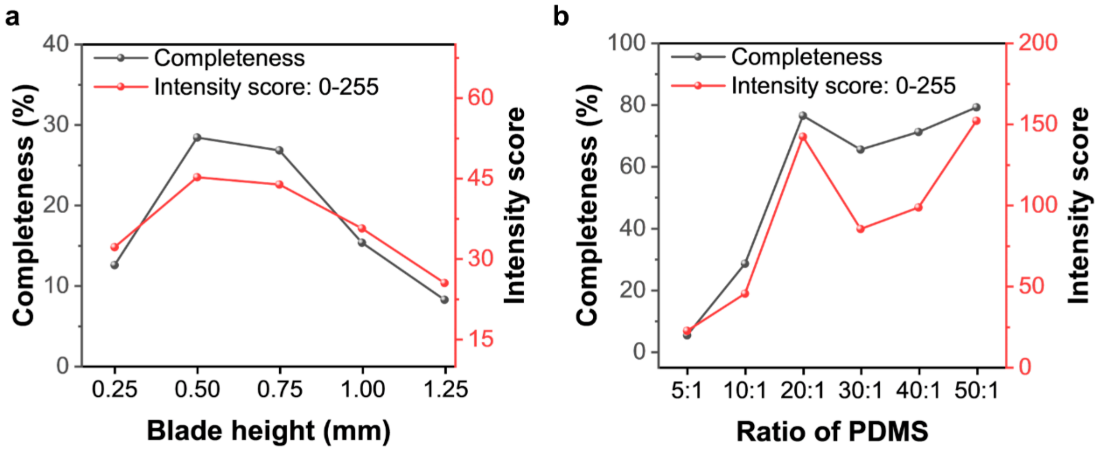

Figure S6. Quantitative TIR image analysis of interfacial contact. (a) Extracted contact completeness (black curve) and optical intensity score (red curve) as a function of blade height, demonstrating optimal geometric conformity at a height of 0.50 mm. (b) Dependence of the contact metrics on the PDMS mixing ratio. The completeness rapidly saturates at a 20:1 ratio, while the optical intensity score exhibits local maxima at 20:1 and 50:1, indicating exceptionally tight localized contact for these specific formulations.

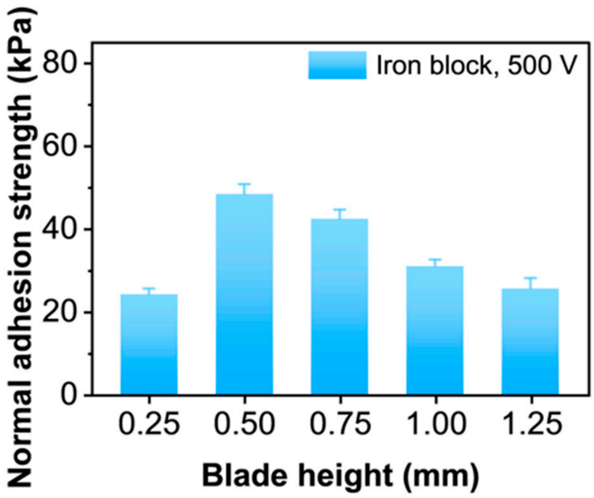

Figure S7. Normal adhesion strength under a 500 V driving voltage (PDMS base: curing agent, 10:1) with the adherend of steel.

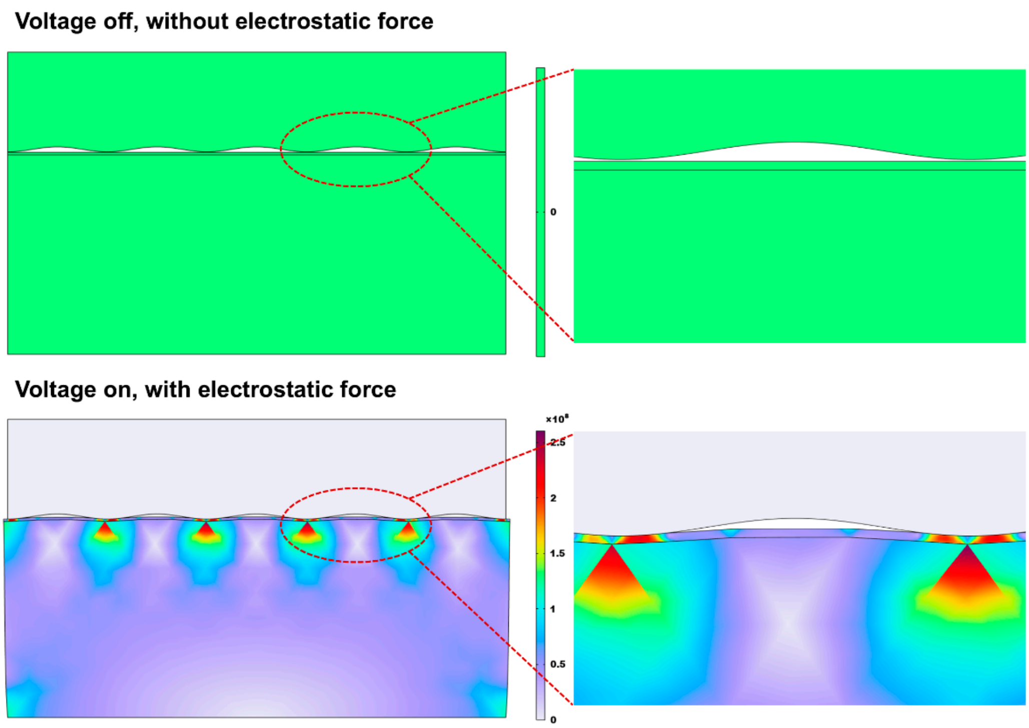

Figure S8. Finite Element Method (FEM) Simulation Analysis of the Electro-induced Conformal Contact Mechanism. **Top:** In the initial state with no applied voltage (Voltage off, no electrostatic force), significant air gaps are evident at the interface due to microscopic roughness. **Bottom:** Upon application of an equivalent electrostatic force (Voltage on), the soft PDMS backing layer undergoes substantial localized elastic deformation under the simulated pressure (indicated by the color-mapped stress field). This underlying elastic deformation effectively drives the conformal adaptation of the top PI dielectric layer to the rough surface, thereby largely eliminating interfacial micro-cavities. **Right:** High-magnification schematic diagrams visually compare the evolution of local microscopic topography and the physical closure of the interface before and after the application of the electrostatic field.

Table S1. Performance comparison of the proposed EA device with low-voltage electroadhesive devices reported in literature.

Ref	Adherend	Working voltage (V)	Normal adhesion strength (kPa)
[17]	PLA	600	0.219
[18]		1000	0.0137
[19]	Glass	800	50.0
[20]	Al	1000	2.13
		800	1.86
		600	1.62
		400	1.07
		200	0.436
	Glass	1000	1.31
		800	1.11
		600	0.872
		400	0.594
		200	0.238
[21]	Al	600	42.5
[22]	Glass	1000	0.200
Our work	Glass	750	113
		500	63.1
		400	41.4

	300	16.4
	200	5.50
	100	1.03
	50	0.400
	25	0.200

Table S2. Surface roughness of both the EA pad and various adherends.

	Sa (μm)	Sq (μm)	Sal (μm)	Sz (μm)
EA device (PI)	0.022	0.036	2.994	0.959
Adherend (Glass)	0.016	0.040	5.606	3.307
Adherend (Steel)	0.134	0.177	3.533	1.908
Adherend (PMMA)	0.014	0.026	4.977	1.329
Adherend (Wood)	5.164	6.722	124.830	49.190
Adherend (A4 paper)	3.603	4.559	73.351	38.487
Adherend (Paperboard box)	0.457	0.582	62.389	7.165
Adherend (Marble)	2.480	3.470	52.116	32.047
Adherend (Alumina ceramic plate)	0.600	0.953	29.090	14.357

Table S3. Actual thicknesses of PDMS backing layers as a function of blade height and PDMS mixing ratio.

Blade height (mm)	Actual PDMS thickness (mm)
0.250 (10:1)	0.115±0.002
0.500 (5:1)	0.283±0.001
0.500 (10:1)	0.285±0.003
0.500 (20:1)	0.282±0.001
0.500 (30:1)	0.294±0.004
0.500 (40:1)	0.268±0.002
0.500 (50:1)	0.294±0.012
0.750 (10:1)	0.437±0.002
1.000 (10:1)	0.577±0.001
1.250 (10:1)	0.691±0.001

Table S4. Electroadhesion force and instantaneous power of EA devices with varying parameters on different adherends at 500 V working voltage. ("10:1-0.25 mm" denotes the preparation parameters of the PDMS backing layer, "10:1" refers to a mixing ratio of 10 parts PDMS base to 1 part curing agent, and "0.25 mm" indicates that the blade height was set to 0.25 millimeters during the blade-coating process.)

EA pad	Adherend	Normal adhesion strength (kPa)	Peak Power consumption (mW)
10:1-0.25 mm	Adherend (Glass)	38.4±4.4	0.1410±0.0000
10:1-0.50 mm	Adherend (Glass)	54.5±1.9	0.1406±0.0003
10:1-0.75 mm	Adherend (Glass)	47.9±3.5	0.1408±0.0003
10:1-1.00 mm	Adherend (Glass)	43.7±0.9	0.1405±0.0003
10:1-1.25 mm	Adherend (Glass)	34.6±3.8	0.1391±0.0002
5:1-0.50 mm	Adherend (Glass)	31.7±1.2	0.1345±0.0004
20:1-0.50 mm	Adherend (Glass)	63.1±1.0	0.1413±0.0005
	Adherend (Steel)	49.5±2.1	0.1320±0.0085
	Adherend (PMMA)	0.0314±0.0177	0.0244±0.0002
	Adherend (Wood)	1.70±0.42	0.0432±0.0003
	Adherend (A4 paper)	4.54±1.62	0.0373±0.0010
	Adherend (Paperboard box)	0.440±0.146	0.0339±0.0002
	Adherend (Marble)	5.98±2.79	0.0361±0.0003
	Adherend (Alumina	0.327±0.129	0.0293±0.0007

	ceramic plate)		
30:1-0.50 mm	Adherend (Glass)	38.6±2.2	0.1416±0.0003
40:1-0.50 mm	Adherend (Glass)	30.5±3.2	0.1408±0.0003
50:1-0.50 mm	Adherend (Glass)	19.7±1.1	0.1398±0.0003

References

17. West, J.; Mici, J.; Jaquith, J.J.; Lipson, H. Design and optimization of millimeter-scale electroadhesive grippers. *J. Phys. D: Appl. Phys.* **2020**, *53*, 435302. https://doi:10.1088/1361-6463/aba1b0

18. Xiang, C.; Guo, J.; Rossiter, J. ContinuumEA: a soft continuum electroadhesive manipulator. In *2018 IEEE International Conference on Robotics and Biomimetics (ROBIO)*; IEEE: Kuala Lumpur, **2018**; pp. 2473–2478. https://doi: 10.1109/ROBIO.2018.8664717.

19. Choi, K.; Kim, S.-H.; Hwang, U.; Kim, J.; Park, I.-K.; Jang, K.-S.; Choi, H.J.; Choi, H.R.; Kim, T.; Suhr, J.; Nam, J.-D. Lifting-Force Maximization of a Micropatterned Electroadhesive Device Comparable to the Human-Finger Grip. *ACS Appl. Electron. Mater.* **2020**, *2*, 1596–1602, https://doi:10.1021/acsaelm.0c00188.

20. Gao, D.; Thangavel, G.; Lee, J.; Lv, J.; Li, Y.; Ciou, J.-H.; Xiong, J.; Park, T.; Lee, P.S. A supramolecular gel-elastomer system for soft iontronic adhesives. *Nat. Commun.* **2023**, *14*, 1990, https://doi:10.1038/s41467-023-37535-4.

21. Mici, J.; Ko, J.W.; West, J.; Jaquith, J.; Lipson, H. Parallel electrostatic grippers for layered assembly. *Additive Manufacturing* **2019**, *27*, 451–460, https://doi:10.1016/j.addma.2019.03.032.

22. Graule, M.A.; Chirarattananon, P.; Fuller, S.B.; Jafferis, N.T.; Ma, K.Y.; Spenko, M.; Kornbluh, R.; Wood, R.J. Perching and takeoff of a robotic insect on overhangs

using switchable electrostatic adhesion. *Science* **2016**, *352*, 978–982, https://doi:10.1126/science.aaf1092.

```
% Quantitative Analysis Script for Interfacial Optical Contact Images (TIR images analysis)
% (For EA Pad Adhesion Analysis)
% Objective: Extract 1. Contact area fraction (Completeness)
% 2. Mean gray value of the contact area (Color Depth)
% 1. Read the original image
img_name = ‘image_569271.jpg’;
original_img = imread(img_name);
% 2. Convert to 8-bit grayscale image
gray_img = rgb2gray(original_img);

% Metric 1: Contact Completenes
% Calculate the global threshold automatically using Otsu’s method
level = graythresh(roi_gray);
% Binarize the image
% (Regions with good contact are darker and typically become 0/black after binarization)
bw_img = imbinarize(roi_gray, level);
% Invert the binarized image so the contact area becomes 1 (white) and background becomes 0 (black)
contact_mask = ~bw_img;
% Calculate the percentage of the effective contact area (Area Fraction)
total_pixels = numel(contact_mask);
contact_pixels = sum(contact_mask(:));
area_fraction = (contact_pixels / total_pixels) * 100;

% Metric 2: Color Depth (Intensity)
% Extract gray values strictly within the “effective contact area” to avoid background interference
```

```
% Gray value ranges from 0 to 255; a lower value indicates a darker region (better optical contact)
contact_gray_values = roi_gray(contact_mask);
mean_gray_value = mean(contact_gray_values);
% Convert to an “Intensity Score” for more intuitive chart visualization
% A higher score represents tighter contact and more thorough air expulsion
intensity_score = 255 - mean_gray_value;

% Result Visualization and Output
figure(‘Name’, ‘Analysis Results’, ‘NumberTitle’, ‘off’);
subplot(1,3,1); imshow(roi_gray); title(‘Original Grayscale ROI’);
subplot(1,3,2); imshow(bw_img); title(‘Otsu Binarization’);
subplot(1,3,3); imshow(contact_mask); title(‘Contact Mask’);
fprintf(‘Effective Contact Area Fraction (Completeness): %.2f %%\n’, area_fraction);
fprintf(‘Mean Gray Value of Contact Area (0-255, lower is better): %.2f\n’, mean_gray_value);
fprintf(‘Optical Contact Intensity Score (0-255, higher is better): %.2f\n’, intensity_score);
```
